# Long Pentraxin PTX3 Exacerbates Pressure Overload–Induced Left Ventricular Dysfunction

**DOI:** 10.1371/journal.pone.0053133

**Published:** 2013-01-23

**Authors:** Satoshi Suzuki, Tetsuro Shishido, Akira Funayama, Shunsuke Netsu, Mitsunori Ishino, Tatsuro Kitahara, Toshiki Sasaki, Shigehiko Katoh, Yoichiro Otaki, Tetsu Watanabe, Yoko Shibata, Alberto Mantovani, Yasuchika Takeishi, Isao Kubota

**Affiliations:** 1 Department of Cardiology, Pulmonology, and Nephrology, Yamagata University School of Medicine, Yamagata, Japan; 2 Department of Cardiology and Hematology, Fukushima Medical University, Fukushima, Japan; 3 Department of Translational Medicine, University of Milan, Milan, Italy; Temple University, United States of America

## Abstract

**Background:**

Left ventricular hypertrophy is enhanced by an inflammatory state and stimulation of various cytokines. Pentraxin 3 (PTX3) is rapidly produced in response to inflammatory signals, and high plasma PTX3 levels are seen in patients with heart failure. This study aimed to examine the influence of PTX3 on cardiac hypertrophy and left ventricular dysfunction with respect to pressure overload.

**Methods and Results:**

PTX3 systemic knockout (PTX3-KO) mice, transgenic mice with cardiac-specific overexpression of PTX3 (PTX3-TG), and the respective wild-type (WT) littermate mice were subjected to transverse aortic constriction (TAC) or a sham operation. Cardiac PTX3 expression increased after TAC in WT mice. In vitro, hydrogen peroxide induced the expression of PTX3 in both cardiac myocytes and cardiac fibroblasts. Recombinant PTX3 phosphorylated extracellular signal–regulated kinase 1/2 (ERK1/2) in cardiac fibroblasts. Phosphorylation of cardiac ERK1/2 and nuclear factor kappa-B after TAC was attenuated in the PTX3-KO mice but was enhanced in the PTX3-TG mice compared with WT mice. Interleukin-6 and connective tissue growth factor production was lower in the PTX3-KO mice than in the WT mice, but this was augmented in the PTX3-TG mice than in the WT mice. Echocardiography revealed that adverse remodeling with left ventricular dysfunction, as well as with increased interstitial fibrosis, was enhanced in PTX3-TG mice, while these responses were suppressed in PTX3-KO mice.

**Conclusion:**

The local inflammatory mediator PTX3 directly modulates the hypertrophic response and ventricular dysfunction following an increased afterload.

## Introduction

Left ventricular hypertrophy (LVH) is an adaptive response of the heart to pressure or volume overload and is recognized as an independent risk factor for cardiovascular disease, congestive heart failure (CHF), and cardiac sudden death [1]. Increasing afterload leads to concentric hypertrophy in order to compensate for the cardiac workload. However, eventually, a persistent hypertrophic state leads to decompensated heart failure, which is characterized by interstitial fibrotic changes, ventricular dilatation, and a progressive decline in cardiac output.

One of the important factors that enhance cardiac remodeling is the presence of an inflammatory state [2]–[4]. Previously, it was reported that interleukin-6 (IL-6) stimulates cardiomyocytes via the gp130 receptor and leads to cardiac hypertrophy and cardiac dysfunction [5], [6]. Increasing plasma levels of inflammatory cytokines in patients with heart failure provide important prognostic information regarding morbidity and mortality [7].

Accumulating evidence suggests that an elevated C-reactive protein (CRP) level is an independent prognostic factor in patients with heart failure [8]. CRP is a classical short pentraxin; pentraxin 3 (PTX3), a member of the long pentraxin family, has a conserved C-terminal pentraxin domain similar to that in classical short pentraxins, but is different in terms of the presence of an unrelated long N-terminal domain [9]. Although CRP is produced in the liver in response to stimulation by various cytokines [10], a variety of cell types, such as dendritic cells, phagocytes, fibroblasts, and endothelial cells, can produce PTX3 on exposure to primary inflammatory signals [11], [12]. We previously reported that plasma PTX3 levels provide important prognostic information for risk stratification of patients with heart failure [13]. However, the effect of PTX3 on pressure overload–induced cardiac hypertrophy is unknown.

In the present study, we used 2 different genotypes of mice—PTX3 systemic knockout (PTX3-KO) mice and transgenic mice with cardiac-specific overexpression of PTX3 (PTX3-TG)—and induced pressure overload in these mice by using the thoracic transverse aortic constriction (TAC) technique. We evaluated cardiac morphology, cardiac function, and cytokine and gene expression in the mice, and examined the role of PTX3 in cardiac remodeling induced by pressure overload.

## Methods

### Ethics Statement

All experimental procedures were performed according to the animal welfare regulations of the Yamagata University School of Medicine, and the study protocol was approved by the Animal Subjects Committee of the Yamagata University School of Medicine. The investigation conformed to the *Guide for the Care and Use of Laboratory Animals* published by the US National Institutes of Health.

### Animal model

PTX3-KO mice were generated as described previously [14]. Human PTX3 cDNA was generated from THP-1, and PTX3-TG mice were created in Yamagata University using standard techniques [15]. The human embryonic lung fibroblast cell line, THP-1 (RCB1189), was provided by the RIKEN BRC through the National Bio-Resource Project of the MEXT, Japan. Briefly, a 5.5-kb fragment of mouse α-MHC gene (a kind gift from Dr J. Robbins, Children's Hospital Research Foundation, Cincinnati, OH) and 1.15 kb human PTX3 cDNA were subcloned into plasmids. Human PTX3 cDNA was generated from THP-1 cells treated with interferon-γ and lipopolysaccharide. The cDNA was amplified with a sense primer (5′-atgcatctccttgcgattctgttttgtgct-3′) and an antisense primer (5′-ttatgaaacatactgagctcctccatccat-3′) designed on the basis of GenBank sequences (NM002852). The plasmid was digested with NotI to generate a DNA fragment composed of the α-MHC gene promoter, PTX3 cDNA, and a poly A tail of the human growth hormone. We microinjected the construct into the pronuclei of single-cell fertilized mouse embryos as previously described [15], [16]. PTX3-KO mice and PTX3-TG mice had a C57BL/6 gene background and a BDF-1 gene background, respectively.

### Experimental protocols

We performed TAC and sham operations on PTX3-KO and PTX3-TG and the respective WT mice [17]. Briefly, eight to ten weeks old male mice were anesthetized by intraperitoneal injection with a mixture of ketamine (80 mg/kg) and xylazine (8 mg/kg). Animals were intubated with a 20-gauge polyethylene catheter and ventilated with a rodent ventilator (Harvard Apparatus, Holliston, MA). The chest cavity was opened at the second intercostal space at the left upper sternal border. The transverse section of the aorta was freed, an 7-0 prolene suture was passed around the aorta between the right innominate and left common carotid arteries, a tight ligature was tied against a 27-gauge needle, and the needle was then promptly removed. In sham animals, the same procedure was performed except for the ligation. To assess morphological changes, 8 mice were weighed for each operation and their hearts and lungs were excised and weighed at 2 and 4 weeks after operation. The heart was embedded in paraffin and sliced serially from the apex to the base. Three sections were stained with hematoxylin-eosin or Masson trichrome stain. Transverse sections were captured digitally, and cardiomyocyte cross-sectional area was measured with the use of a Scion imaging system (Version 4.0, Scion Corporation, Frederick MD) as previously reported [17]. At least 200 cardiomyocytes were examined in each heart, and the data were averaged. To assess the degree of fibrosis, the section stained with Masson trichrome were scanned with computer-assisted videodensitometry, and the images from at least 20 fields for each heart were analyzed as previously reported [17], [18].

### Echocardiography

To evaluate cardiac function, we performed transthoracic echocardiography using an FFsonic 8900 equipped with a 13-MHz phased-array transducer (Fukuda Denshi Co, Tokyo, Japan) [19], [20]. Mice were lightly anesthetized with intra peritoneal injection of pentobarbital sodium (35 mg/kg), maintaining heart rate at 450–500 beats/min, at 2 and 4 weeks after TAC or sham operation. The heart was imaged in the two-dimensional mode in the parasternal short-axis view, and M-mode image was traced from this view. Interventricular septum (IVS), left ventricular posterior wall (LVPW) thickness and internal dimensions at end-diastole (LVEDD) and end-systole (LVESD) were measured digitally on the M-mode tracings and averaged for 3 cardiac cycles. Left ventricular fractional shortening (FS) was calculated using the formula %FS = (LVEDD – LVESD)/(LVEDD)×100 [16], [19].

### Immunohistochemistry

To examine whether the PTX3 was produced from the heart tissue, myocardial sections were treated with primary anti-PTX3 antibody (1∶100) (Perseus Proteomics Inc, Tokyo, Japan) at 4°C for overnight, subsequently, sections were treated with mouse secondary antibody for 10 minutes and coloration was enhanced with 3,3′-diaminobenzidine, according to the guide for commercially available kit (Histofine® MOUSESTAIN KIT, Nichirei Bioscience, Tokyo, Japan).

### Western Blotting

Total protein was extracted from the snap-frozen left ventricle with ice-cold lysis buffer containing 20 mM Tris-HCl (ph 7.5), 150 mM NaCl, 1 mM Na2EDTA, 1 mM EGTA, 1% Triton, 2.5 mM sodium pyrophosphate, 1 mM β-glycerophosphate, 1 mM Na3VO4, 1 µg/ml leuptin. Nuclear extracts were obtained by using NE-PER nuclear and cytoplasmic extraction reagent (Thermo scientific, Rockford, IL). Equal amounts of total protein and nuclear extracts were separated on each gel lane of 10% SDS-PAGE and electroblotted onto polyvinylidene difluoride membrane (Hybond P, Amersham Bioscience Corp. Piscataway, NJ). Rabbit polyclonal antibodies for p38 MAP kinase, phospho-p38 MAP kinase, extracellular signal-regulated kinase1/2 (ERK1/2), phospho-ERK1/2, c-Jun N-terminal kinase (JNK), phospho-JNK (Cell Signaling Technology, Danvers, MA), mice monoclonal antibody of PTX3 (Proteomics Inc, Tokyo, Japan) and goat anti-β-actin antibody (SIGMA, Saint Louise, MO) were used. For nuclear proteins, membranes were incubated with specific anti-nuclear factor kappa B (NF-κB) p65 rabbit polyclonal antibody (Santa Cruz Biotechnology) [21]. Changes in these MAP kinase and NF-κB levels with respect to control values (wild-type sham) were estimated and the results were expressed as arbitrary units.

### Extraction of Total RNA and Quantitative Real-time Reverse Transcriptase-PCR

Total protein and mRNAs were extracted from the snap-frozen left ventricle for western blotting analysis and real-time reverse transcriptase-PCR (RT-PCR) amplification using a LightCycler DNA Master SYBR Green I (Roche Diagnostics, Tokyo, Japan). Data are expressed as fold increase over the values for sham-operated WT mice. Primers were designed on the basis of GenBank sequences (atrial natriuretic factor (ANF), K02781; β-myosin heavy chain (MHC), AY056464; α-MHC, M76601; collagen type I, NM007742; collagen type III, NM009930; connective tissue growth factor (CTGF), NM010217; IL-6, NM031168; and GAPDH, NM001001303).

### Enzyme-linked immunosorbent assay of TNF-α and IL-6

Mouse blood was collected from the tail into the plasma separation tube at 5 days after TAC and immediately centrifuged. Plasma levels of IL-6 were measured by a commercially available ELISA kit (Mouse IL-6 Immunoassay, R&D Systems, Minneapolis, MN) [2].

### Neonatal cardiac myocytes and fibroblasts culture

We performed in vitro study to investigate the cellular source of PTX3 production using neonatal rat cardiac myocytes and cardiac fibroblasts [16], [19]. Briefly, ventricles were obtained from 1- or 2-day-old Sprague-Dawley rats, and cardiac myocytes and cardiac fibroblasts were isolated by collagenase digestion. Non-cardiomyocytes were removed removed via 2 rounds of preplating on culture dishes and fibroblasts were obtained from preplating culture dishes. Cell cultures of cardiac myocytes and cardiac fibroblasts were treated with hydrogen peroxide (H_2_O_2_) for 6 hours, and the expression of PTX3 was evaluated by Western blotting analysis as described above using anti-PTX3 antibody (Santa Cruz Biotechnology, Santa Cruz, CA). To determine the effect of PTX3 on cardiac remodeling in vitro, cardiac myocytes and cardiac fibroblasts were incubated with recombinant human PTX3 reagents (100 nM) for 15 minutes, thereafter, protein extracts were collected for Western blotting analysis.

### Statistical Analysis

All results are expressed as mean ± standard deviation (SD). Effects of TAC on heart and lung weight, histological data, echocardiographic data, hemodynamic data, western blotting data, and RT-PCR data were compared between the respective WT mice. Gene-targeted mice were analyzed using one-way analysis of variance (ANOVA) followed by multiple comparisons using Scheffe's test. A *P* value less than 0.05 was considered statistically significant. Statistical analysis was performed using a standard statistical program package (Stat View; version 5.0, SAS Institute Inc., Cary, NC).

## Results

### PTX3 expression induced after TAC

**Figure 1:**
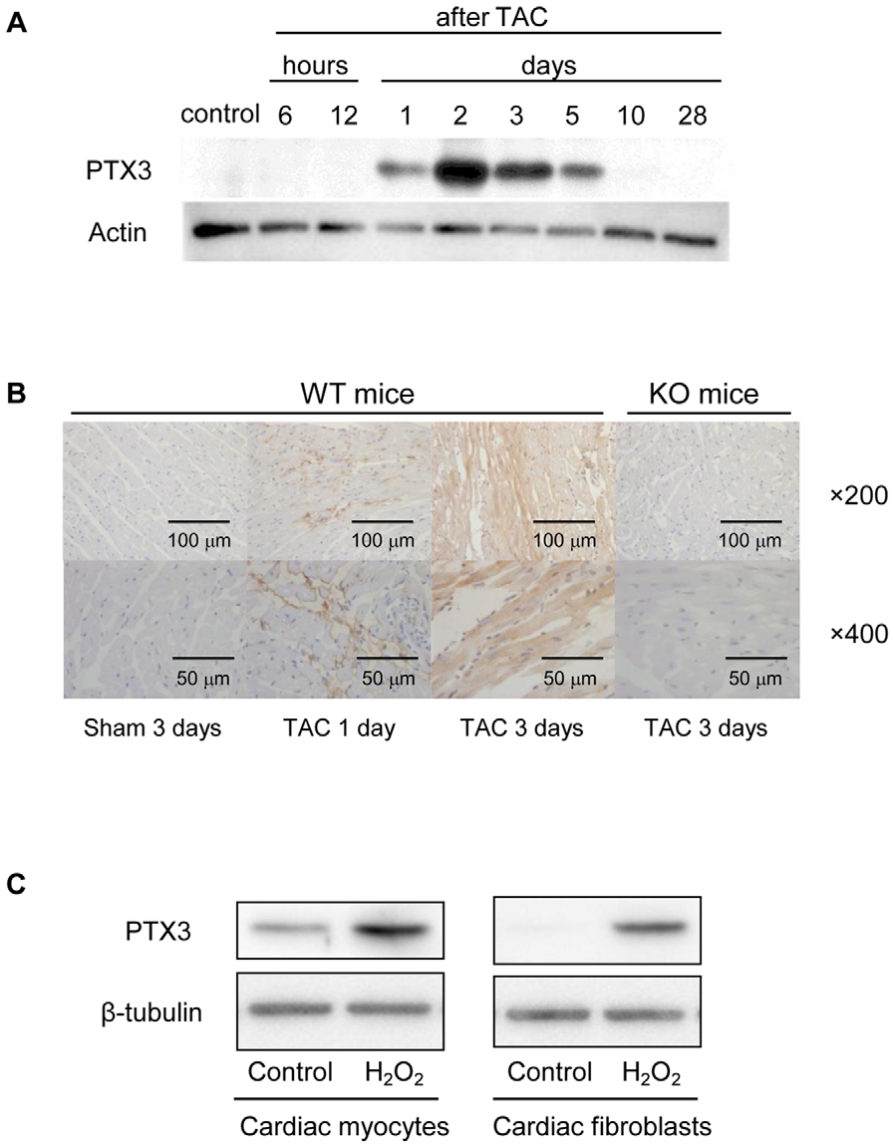
Increase in PTX3 expression after TAC operation and H_2_O_2_ stimulation. A. Time course of PTX3 expression in heart tissue after TAC. B. Immunohistochemistry of heart samples after TAC operation using anti PTX3 antibody. (top; ×200, bottom; ×400) C. PTX3 expression after H_2_O_2_ stimulation using neonatal rat cardiac myocytes and cardiac fibroblasts (left panel; cardiac myocytes, right panel; cardiac fibroblasts).

Since increased plasma levels of PTX3 in patients with heart failure have been previously demonstrated [13], we examined whether PTX3 was produced by heart tissue after the TAC surgery. As expected, expression of PTX3 by hearts experiencing pressure overload was increased at 1–10 days after TAC; in particular, a large amount of PTX3 was produced at 2–3 days (Figure 1A). We performed immunohistochemical staining of the heart after TAC in PTX3 KO mice and WT mice (Figure 1B). Although the heart was not stained with anti-PTX3 antibody in TAC operated PTX3-KO mice and sham operated WT mice, PTX3 was produced from the non-cardiomyocyte at 1 day after TAC, and from cardiac myocytes at 3 days after TAC in WT mice. We attempted additional in vitro study to examine what the main cellular source for PTX3 production in response to cellular damage is using neonatal rat cardiac myocytes and fibroblasts. In cardiac myocytes, we found that the expressions of PTX3 were increased after H_2_O_2_ stimulation (Figure 1C). Similarly, the enhancement of PTX3 expressions were more potentiated in fibroblasts after H_2_O_2_ stimulation (Figure 1C).

### PTX3 activated mitogen-activated protein (MAP) kinases and nuclear factor-kappa B (NF-κB) after TAC

**Figure 2:**
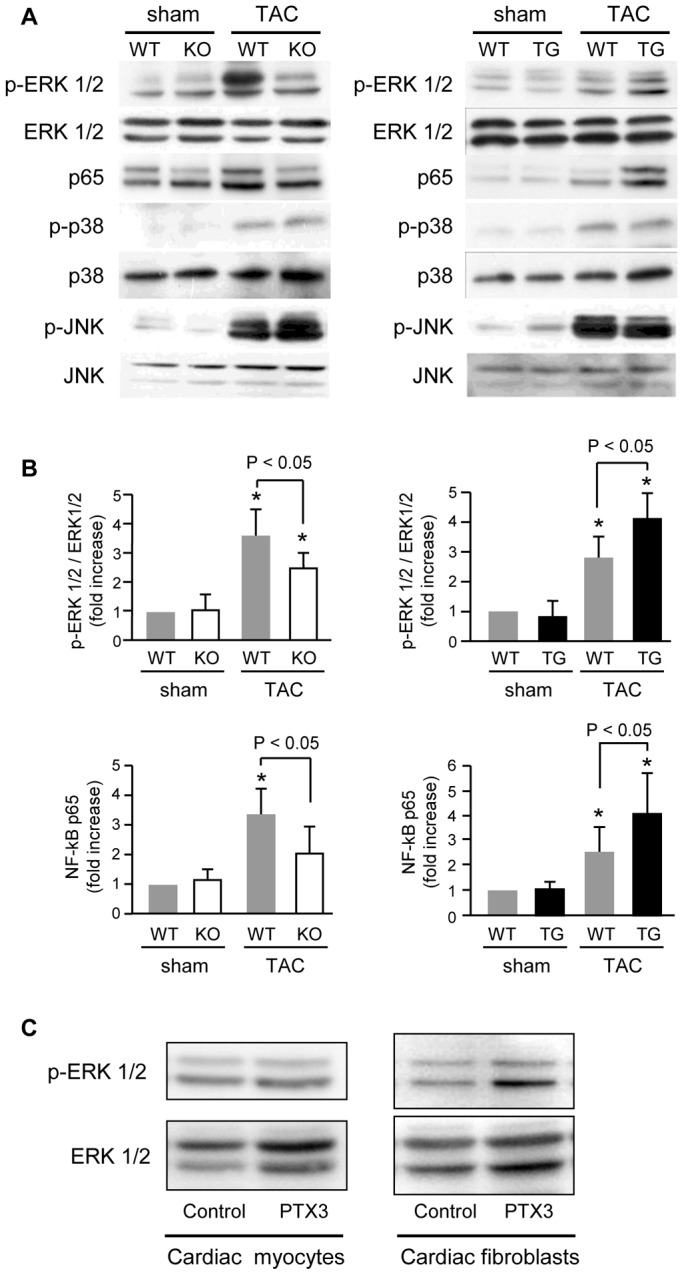
Phosphorylations of MAP kinase and nuclear transcriptional factor, NF-κB after TAC operation. A. Increase in phosphorylations of MAPK and NF-κB after TAC operation in PTX3-KO mice (left panel) and in PTX3-TG mice (right panel). B. Modulation of ERK1/2 and NF-κB p65 phosphorylation in PTX3-KO (left), PTX3-TG (right), compared with WT mice. C. ERK1/2 phosphorylation after recombinant PTX3 stimulation (left panel; cardiac myocytes, right panel; cardiac fibroblasts). *P<0.01 vs. sham-operated mice of the same strain. Results are expressed as mean ± SD (n = 8).

Next, we evaluated the activities of MAP kinase and NF-κB after TAC in PTX-3 KO and PTX3-TG mice (Figure 2A), since recent evidence has suggested that CRP enhances cell proliferation and tissue factor expression in VSMC and EC by binding with the Fc gamma receptor as well as the activation of the MAP kinase family, especially ERK1/2 [9], [22]. We found that the ERK1/2 and NF-κB p65 were phosphorylated 5 days after TAC; however, those in PTX3-KO mice were lower than that in WT mice (*P*<0.05) (Figure 2A and B). Although phosphorylation of p38 MAP kinase and c-Jun N-terminal kinase increased similarly after TAC, no difference was observed between the phosphorylation levels in PTX3-KO and WT mice (Figure 2A). In contrast, phosphorylation of ERK1/2 and NF-κB p65 in PTX-TG mice was greater than that in WT mice (Figure 2B). Cardiac myocytes and cardiac fibroblasts isolated from the neonatal rat heart were incubated with 100 nM recombinant human PTX3 reagents for 1 hr (R&D systems, Inc. Minneapolis, MN.). As shown in Figure 2C, ERK1/2 was phosphorylated by PTX3 in fibroblast, but not in cardiac myocytes, suggesting that hypertrophic signaling in response to PTX3 was more sensitive in fibroblasts than in cardiac myocytes.

### Expression of inflammatory cytokines after TAC

**Figure 3:**
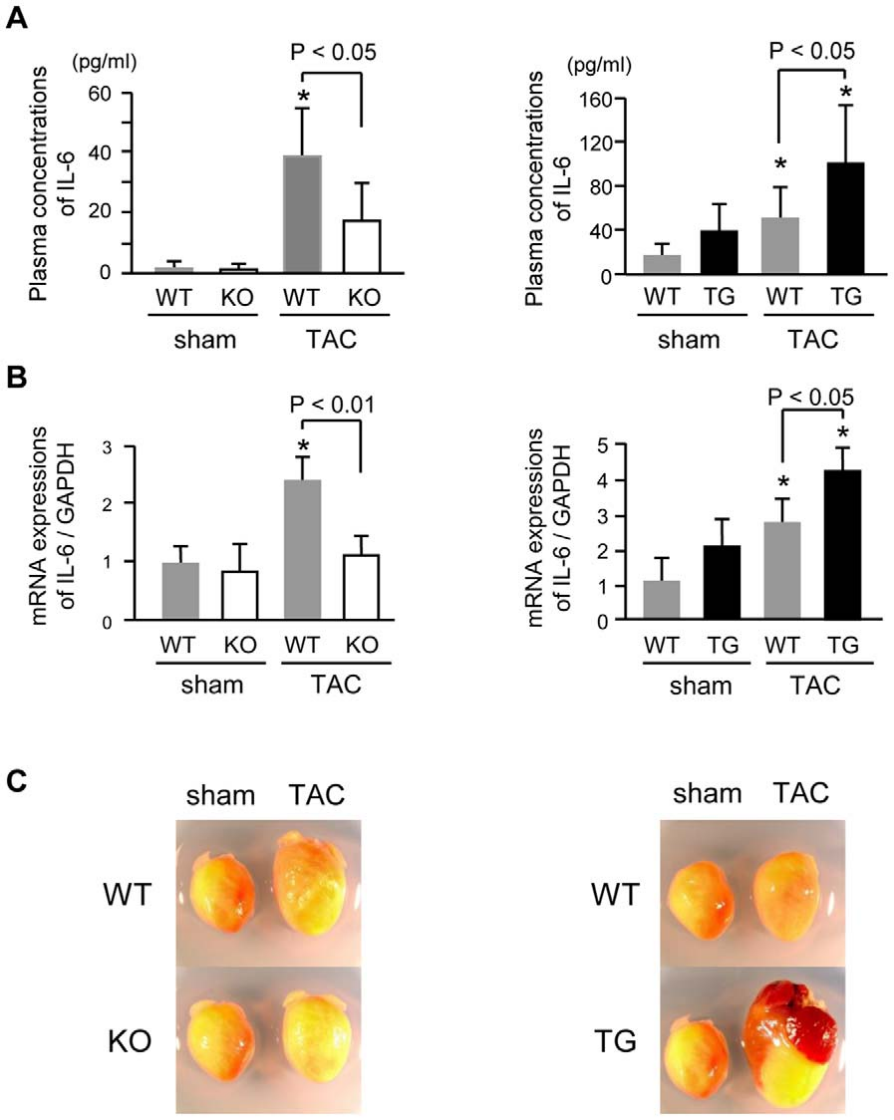
Hypertrophic changes after TAC operation. A. Plasma concentrations of IL-6 at 5 days after TAC or sham operation in PTX3-KO mice (left) and PTX3-TG mice (right). B. IL-6 expression after TAC detected by quantitative PCR in PTX3-KO mice (left) and PTX3-TG mice (right). C. Representative photographs of hearts at 4 weeks after TAC and sham operation in PTX3-KO mice (left) and PTX3-TG mice (right). *P<0.01 vs. sham mice of the same strain. Results are expressed as mean ± SD (n = 6–8).

We evaluated the degree of systemic inflammation in the plasma and heart. Plasma IL-6 concentrations (Figure 3A) and myocardial IL-6 mRNA expression (Figure 3B) in TAC-operated mice were significantly higher than those in sham-operated mice, but were lower in TAC-operated PTX3-KO mice and much higher in PTX3-TG mice than in the respective WT mice, suggesting that PTX3 may modulate ventricular remodeling after TAC.

### Myocardial hypertrophic changes after TAC

**Figure 4:**
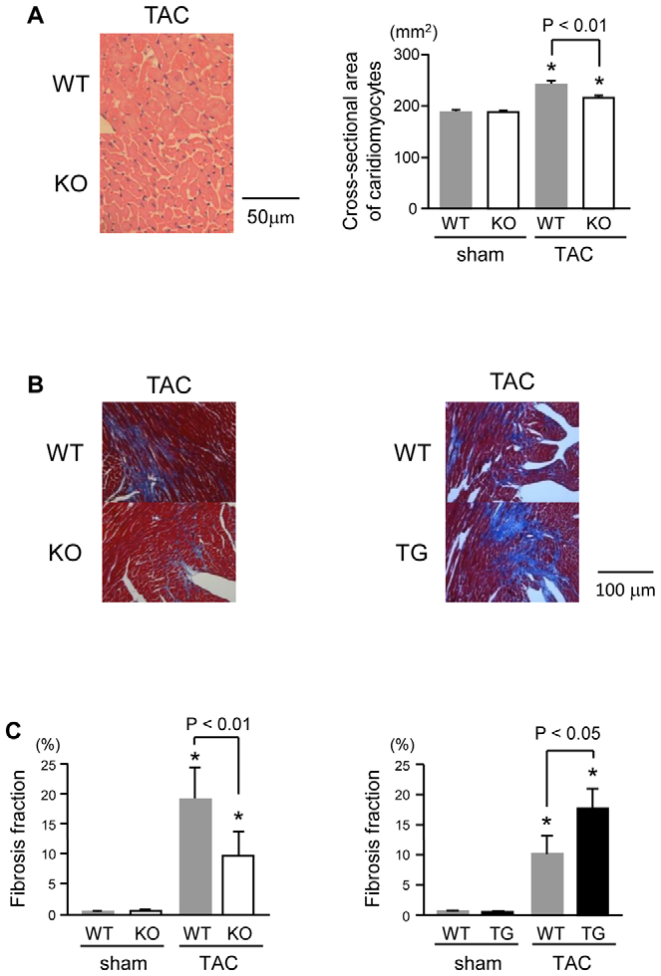
Hypertrophic and fibrotic changes after TAC operation. A. Representative histological micrographs of hematoxylin-eosin-stained and quantitative analysis of the cross-sectional areas of cardiomyocytes. B. Masson's trichrome-stained sections of the left ventricular myocardium in PTX3-KO mice strain (left) and in PTX3-TG mice strain (right) at 2 weeks after TAC or sham operation. C. Quantitative analysis of the fibrosis fractions at 2 weeks after TAC or sham operation, respectively (n = 8). *P<0.01 vs. levels in sham-operated mice of the same strain. Results are expressed as mean ± SD (n = 8).

The ratio of heart weight to body weight (HW/BW) was significantly increased after TAC in both WT mice and PTX3-KO mice, and was significantly lower in PTX3-KO mice than in WT mice at 4 weeks after TAC (Table 1). On the contrary, the HW/BW ratio was higher in PTX3-TG mice than in WT mice at 4 weeks after TAC (*P*<0.01) (Table 2). PTX3-KO mice exhibited a significantly lower lung weight to body weight ratio (LW/BW) than WT mice after TAC (Table 1); these values were significantly higher in PTX3-TG mice than in the WT mice after TAC (Table 2). Similarly, microscopic analysis demonstrated that the cross-sectional areas of cardiomyocytes were increased in both WT and PTX3-KO mice at 2 weeks after TAC, but this increase was significantly attenuated in PTX3-KO mice as compared with WT mice (P<0.01) (Figure 4A).

**Table 1:** Gravimetric and echocardiographic data of PTX3-KO mice after TAC.

	Sham	TAC 2 weeks	TAC 4 weeks
**Gravimetric data**			
**BW (g)**			
WT	24.9±1.2	22.9±2.0	22.5±2.3*
PTX3-KO	24.3±1.5	23.8±1.6	23.4±2.0
**HW to BW ratio (mg/g)**			
WT	4.51±0.10	7.00±1.33**	7.92±1.44**
PTX3-KO	4.73±0.52	5.49±0.34* #	6.24±0.51* #
**LW to BW ratio (mg/g)**			
WT	5.14±0.41	7.46±1.91**	8.12±1.94**
PTX3-KO	5.10±0.64	5.72±0.49#	5.30±0.34#
**Echocardiographic data**			
**Heart rate (beats/min)**			
WT	506±24.1	512±30.4	479±39.1
PTX3-KO	497±16.7	499±21.0	464±30.4
**IVS thickness (mm)**			
WT	0.73±0.08	1.03±0.09**	1.14±0.09**
PTX3-KO	0.70±0.06	0.88±0.08* #	0.90±0.07* #
**LVEDD (mm)**			
WT	3.07±0.14	2.59±0.23	3.19±0.34
PTX3-KO	2.95±0.15	2.65±0.27	2.69±0.11#
**LVFS (%)**			
WT	48.5±2.2	47.0±5.0	40.4±4.4**
PTX3-KO	48.7±1.4	52.0±3.8	51.0±4.7#

WT, Wild type; PTX3-KO, PTX3 systemic knockout mice; TAC, thoracic aortic constriction; BW, body weight; HW, heart weight; LW, lung wet weight; IVS, interventricular septal wall thickness; LVEDD, left ventricular end-diastolic diameter; LVFS, left ventricular fractional shortening. All data are shown as mean ± SD (n = 10 per group).

* *P*<0.05 and

** *P*<0.01 vs. sham-operated mice of the same strain;

# *P*<0.05 and

## *P*<0.01 vs. TAC-operated WT mice at each time point, respectively.

**Table 2:** Gravimetric and echocardiographic data of PTX3-TG mice after TAC.

	Sham	TAC 4 weeks
**Gravimetric data**		
**BW (g)**		
WT	27.2±1.7	25.3±3.8*
PTX3-TG	28.0±1.8	24.2±2.5*
**HW to BW ratio (mg/g)**		
WT	5.10±0.22	6.78±0.76**
PTX3-TG	5.07±0.31	8.03±1.68** #
**LW to BW ratio (mg/g)**		
WT	5.21±0.61	6.22±0.73*
PTX3-TG	5.21±0.49	9.28±3.17** #
**Echocardiographic data**		
**Heart rate (beats/min)**		
WT	501±21.2	486±33.7
PTX3-TG	509±19.4	483±47.3
**IVS (mm)**		
WT	0.67±0.05	1.00±0.18**
PTX3-TG	0.67±0.07	0.72±0.14##
**LVEDD (mm)**		
WT	2.88±0.20	2.82±0.25
PTX3-TG	2.96±0.14	3.54±0.31##
**LVFS (%)**		
WT	51.6±4.3	44.6±3.8**
PTX3-TG	51.7±4.6	35.5±2.7** ##

Abbreviations as in Table 1. PTX3-TG, PTX3 cardiac-specific overexpression mice. All data are shown as mean ± SD (n = 10 per group,).

* *P*<0.05 and

** *P*<0.01 vs. sham mice of the same strain;

# *P*<0.05 and

## *P*<0.01 vs. TAC-operated WT mice at each time point, respectively.

### Myocardial fibrosis and profibrotic genes after TAC

**Figure 5:**
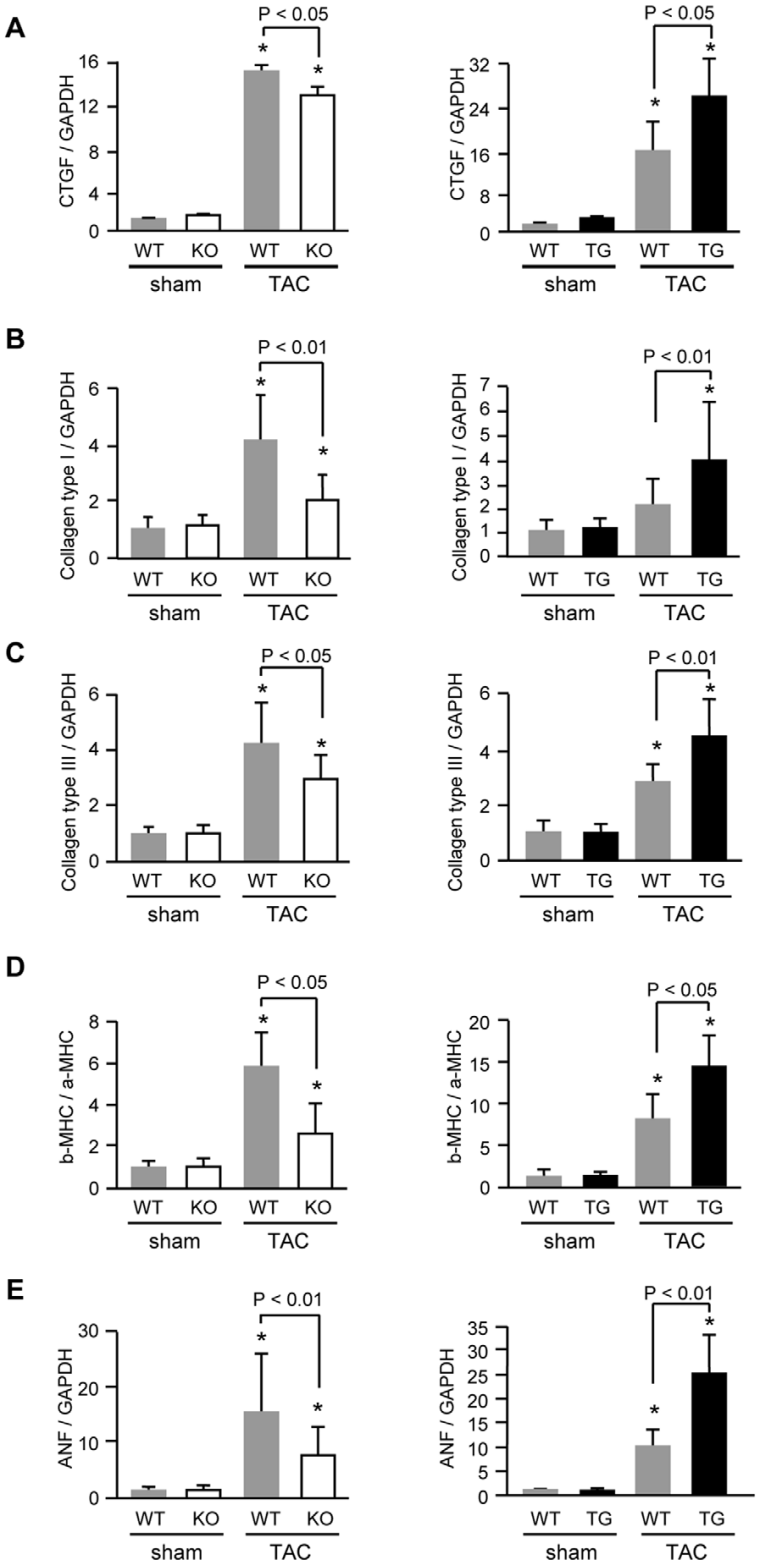
Quantitative analysis of profibrotic and fetal type genes expression by real-time PCR in PTX3-KO (left) and in PTX3-TG (right) at 2 weeks after TAC or sham operation (n = 8). A, CTGF; B, collagen type I; C, collagen type III; D, β- MHC to α-MHC ratio; and E, ANF. Each expression levels was normalized to the GAPDH levels and expressed as fold increase over the levels in the respective WT sham-operated mice. *P<0.01 vs. expression in sham mice of the same strain. Results are expressed as mean ± SD.

Expression of CTGF by activation of NF-κB enhances extracellular matrix production [23], [24]. To confirm the effect of PTX3 on interstitial fibrosis, we performed a quantitative analysis of Masson's trichrome-stained sections (Figure 4B). The degree of myocardial fibrosis after TAC was attenuated in PTX3-KO mice than in the respective WT mice (*P*<0.01) (Figure 4C). We also observed that mRNA expression of CTGF (Figure 5A), collagen type I (Figure 5B), and collagen type III (Figure 5C) were attenuated in PTX3-KO mice. In PTX3-TG mice, the degree of myocardial fibrosis and cardiac fibrosis-related gene expression were markedly higher than in the WT mice (Figure 4B, C, 5A, B).

### Cardiac function and induction of fetal genes after TAC

Excessive myocardial fibrosis and hypertrophy in response to pressure overload aggravates cardiac dysfunction by reducing pumping activity [4], [25]. The left ventricular (LV) end-diastolic diameter (LVEDD) was markedly increased after TAC, but significantly smaller in PTX3-KO mice than in WT mice. Fractional shortening (FS) was also better preserved in PTX3-KO mice than in WT mice (Table 1). We quantified the mRNA expression of fetal-type genes by using real-time RT-PCR. The β-MHC/α-MHC ratio (Figure 5D) and gene expression of atrial ANF (Figure 5E) in response to TAC were significantly attenuated in PTX3-KO mice compared to WT mice. In contrast, PTX3-TG mice after TAC showed larger LVEDD and lower LVFS than WT mice (Table 2), as well as an increase of the β-MHC/α-MHC ratio and ANF expression in PTX3-TG mice (Figure 5D, E).

## Discussion

In the present study, we describe the importance of PTX3 on cardiac ERK1/2 activation and cytokine expression as well as on subsequent cardiac dysfunction. PTX3 expression increased in the heart after pressure overload and in cardiomyocyte and fibroblasts under hydrogen peroxide stimulation. Recombinant PTX3 induced ERK1/2 phosphorylation in cardiac fibroblasts. In vivo study, we found that PTX3 deficiency significantly reduced ERK1/2 and p65 activation and cytokine expression. Furthermore, we demonstrated that cardiac hypertrophy, fibrosis, and dysfunction after pressure overload were significantly attenuated in PTX3-KO mice than in WT mice. Notably, PTX3-TG mice showed higher levels of IL-6 expression, cardiac fibrosis, and cardiac dysfunction. These data provide the first direct evidence of the involvement of PTX3 expression in cardiac remodeling after pressure overload.

Salio et al reported that PTX3 staining was only extracellular after acute myocardial ischemia in animal model [14]. In contrast, Peri, et al. reported that myocyte sarcoplasma was strongly stained by PTX3 antibody in human autopsy heart samples after myocardial infarction [26]. This discrepancy might be owing to the differences in the degree of myocardial damage, duration of ischemia, species, and antibody used. Although, Peri, et al. studied autopsy myocardial samples from patients who died after acute myocardial infarction, Salio, et al. used acute ischemic heart samples obtained from living mice until receiving euthanasia. Therefore, it is possible that physiological myocardial stress might be different between two studies. Another study by Introna, et al. showed that PTX3 positive cells were distributed throughout the heart using in situ hybridization [27]. In addition, we performed immunohistochemical staining of the heart and clearly demonstrated that PTX3 expression was seen in non-cardiomyocyte firstly, and seen mainly in cardiomyocytes at 3 days after TAC (Figure 1B). Moreover, the heart tissue of TAC operated PTX3-KO mice was not stained at all with anti-PTX3 antibody. This discrepancy between studies by Salio, et al. and ours might be possibly explained by the difference in animal models: acute ischemia vs. chronic pressure overload. Taken together, although PTX3 expression was extracellular in a mouse model of acute myocardial ischemia, we clearly showed by immunohistochemical staining that PTX3 expression was observed in cardiac fibroblasts and subsequently cardiac myocytes in mice during pressure overload.

Several reports have reported the association of inflammation with the development of cardiac failure [28]–[30]. Although Salio et al. have shown that cardiac PTX3 expression is increased during ischemia reperfusion injury [14], the role of PTX3 on cardiac function after pressure overload has not been reported. Recent evidence has demonstrated that both short pentraxin, CRP, and long pentraxin, PTX3, modulate inflammation in several cells, including endothelial cells, smooth muscle cells, and fibroblasts [9], [21], [31], Moreover, Nagai et al. recently reported that cardiac-specific overexpression of human CRP induces cardiac remodeling, including de-compensated heart failure and cardiac fibrosis, in response to pressure overload [32]. In the present study, we found that PTX3 expression was increased in vivo and in vitro study. Moreover, we demonstrated that PTX3-TG mice exhibited a greater extent of cardiac fibrosis and severe cardiac dysfunction than WT mice. Importantly, we also showed that cardiac dysfunction after TAC was attenuated in PTX3-KO mice, suggesting that similar mechanisms in which CRP induces cardiac remodeling may be associated with PTX3-dependent cardiac dysfunction. In the present study, cardiac hypertrophy was significantly attenuated in PTX3-KO mice than in WT mice at 2 weeks after TAC operation, however, fractional shorting was not change in this period. Since we found that cardiac dysfunction was attenuated in PTX3-KO mice at 4 weeks after TAC operation compared with WT mice, these findings indicated that TAC induced PTX3 expressions modulate LV hypertrophic remodeling, and result in accelerated cardiac dysfunction.

Both CRP and PTX3 can bind to the Fc gamma receptor [9], [33] to affect activation of MAP kinases, ERK1/2, and NF-κB [21], [22] proteins, which play critical roles in cardiac remodeling [34], [35]. CRP increases the expression of reactive oxygen species, cytokines, and chemokines (IL-6, monocyte chemoattractant protein-1, and inducible nitric oxide synthase) [22], [31], [36]. We examined the effect of PTX3 on cardiac remodeling after pressure overload and found that phosphorylation of ERK1/2 and NF-κB and expression of IL-6, CTGF, collagen type I and III, and fetal cardiac genes were inhibited by deletion of PTX3 and were enhanced in PTX3-TG mice, similar to CRP transgenic mice. As shown in Figure 2C, ERK1/2 was phosphorylated by PTX3 in cardiac fibroblasts, suggesting that hypertrophic signaling in response to PTX3 was more sensitive in fibroblasts than in cardiac myocytes. Since stimulated cardiac fibroblasts increase the production of cytokines and growth factors [23], [37], [38], which modulates intracellular signaling of cardiac myocytes and leads to induce exacerbation of cardiac hypertrophy and dysfunction during pressure overload. This finding indicates that PTX3 might enhance cardiac responses to pressure overload. This was supported by our present results that IL-6 and CTGF expression and the degree of fibrotic change were suppressed in PTX3-KO mice and were increased in PTX3-TG mice compared with respective WT mice after pressure overload (Figures 3A, 3B, 4B, 4C, and 5A).

A recent study reported that PTX3-KO mice undergoing coronary artery ligation and reperfusion showed greater no-reflow areas and increased neutrophil infiltration [14]. However, Souza et al. demonstrated that PTX3 enhances NF-κB activation and increases ischemia reperfusion injury [21], They explained this inconsistency by suggesting that inflammation is tightly restricted to the heart, but intestinal I/R is widely expressed systemically. We demonstrated that IL-6 circulation and production in the heart were increased by PTX3. IL-6 also activates ERK1/2 through the gp130 receptor and promotes cardiac hypertrophy, but inhibits cardiomyocyte apoptosis [5]; increasing IL-6 levels may also accelerate the hypertrophic response in addition to the direct effect of PTX3. Moreover, inflammatory activation aggravates cardiac fibrosis after TAC operation [30]. These results strongly suggest that PTX3 accelerates the inflammatory response and LV remodeling after pressure overload.

There are several limitations in this study. In the present study, we examined cardiac phenotype of PTX3-KO mice in comparison with WT mice of same strain. Similarly, PTX3-TG mice were compared with WT mice of same background. We found that cardiac hypertrophic remodeling at 4 weeks after TAC was worsen in PTX3-TG mice than in WT mice, but not further aggravated compared with PTX3-KO strain. It is possible that different degree of hypertrophy after TAC was attributed to the difference of mice strain in TG and KO mice. Moreover, the BW was smaller in KO mice strain than in TG mice strain at base line (Table 1 and 2). Several reports have shown that there are large strain-dependent differences in TAC or training responses among mouse strains [39], [40]. These differences might influence the degree of phosphorylation of ERK1/2 in WT mice of PTX3-KO strain and in WT mice of PTX3-TG mice. Moreover we found similar results showing that PTX3 worsened the degree of cardiac fibrosis and dysfunction after pressure overload compared with each strain-matched WT mice. Therefore, the findings of this study imply that PTX3 could be a therapeutic target in patients with cardiac dysfunction.

In the present study, we demonstrated that PTX3 deteriorates LV function and accelerates decompensated diastolic changes through ERK1/2 and NF-κB activation under pressure overload. Inhibition of PTX3 may be a therapeutic target for preventing the development of maladaptive myocardial remodeling in response to pressure overload.
